# Advances in Decoding Bacterial N-Terminal Proteoforms: Technologies, Challenges, and Functional Insights

**DOI:** 10.3390/microorganisms14081671

**Published:** 2026-07-30

**Authors:** Valdes Snauwaert, Petra Van Damme

**Affiliations:** iRIP Unit, Laboratory of Microbiology, Department of Biochemistry and Microbiology, Ghent University, 9000 Ghent, Belgium; valdes.snauwaert@ugent.be

**Keywords:** alternative translation initiation, genome engineering, multiplexed recombineering, N-terminal proteoforms, N-terminomics, riboproteogenomics

## Abstract

The bacterial proteome is a highly dynamic landscape rather than simply a static reflection of the genome. Recent research has revealed that proteome complexity extends far beyond canonical gene annotation, with N-terminal (Nt-)proteoforms emerging as an important underexplored additional regulatory layer. These molecular variants originate from a single genetic locus through alternative translation initiation at internal or external in-frame start sites, thereby generating N-terminal heterogeneity that can influence protein stability, subcellular localization, interaction networks, and the stoichiometric assembly of multiprotein complexes. While recent advances in riboproteogenomics, N-terminomics, and computational annotation strategies have enabled proteoform mapping at single-amino-acid resolution, rapid high-throughput discovery currently outpaces downstream functional characterization. This review discusses the technological advances driving Nt-proteoform discovery, including emerging ribosome profiling and proteogenomic approaches, and further evaluates strategies for the functional characterization of Nt-proteoforms. Particular emphasis is placed on the transition from conventional plasmid-based heterologous expression systems towards precise genome-engineering approaches that enable selective manipulation of alternative translation initiation events within their native genomic context. Such targeted strategies are essential to bridge the gap between Nt-proteoform identification and functional understanding, ultimately uncovering how individual bacterial genomic loci can encode proteoforms with distinct and potentially divergent functional roles in bacterial physiology and pathogenesis. Ultimately, we hypothesize that alternative translation initiation represents a biologically meaningful post-transcriptional regulatory mechanism that contributes to maximizing prokaryotic coding capacity without expanding genome size, rather than merely constituting stochastic translational noise.

## 1. N-Terminal Proteoforms Expand Bacterial Proteome Complexity

The central dogma of molecular biology, originally proposed by Francis Crick, describes a unidirectional flow of genetic information from DNA through RNA to functional proteins [1]. However, the discovery of alternative splicing by Sharp and Roberts demonstrated that eukaryotic gene sequences are often non-contiguous, thereby challenging the presumed linear relationship between genes and their protein products. More recently, the identification of defense-associated reverse transcriptases (DRTs) capable of synthesizing alternating poly(GT/AC) double-stranded DNA (dsDNA) revealed a protein-templated mechanism for sequence-specific DNA synthesis, further expanding the conceptual boundaries of information flow in biology [2].

**Figure 1:**
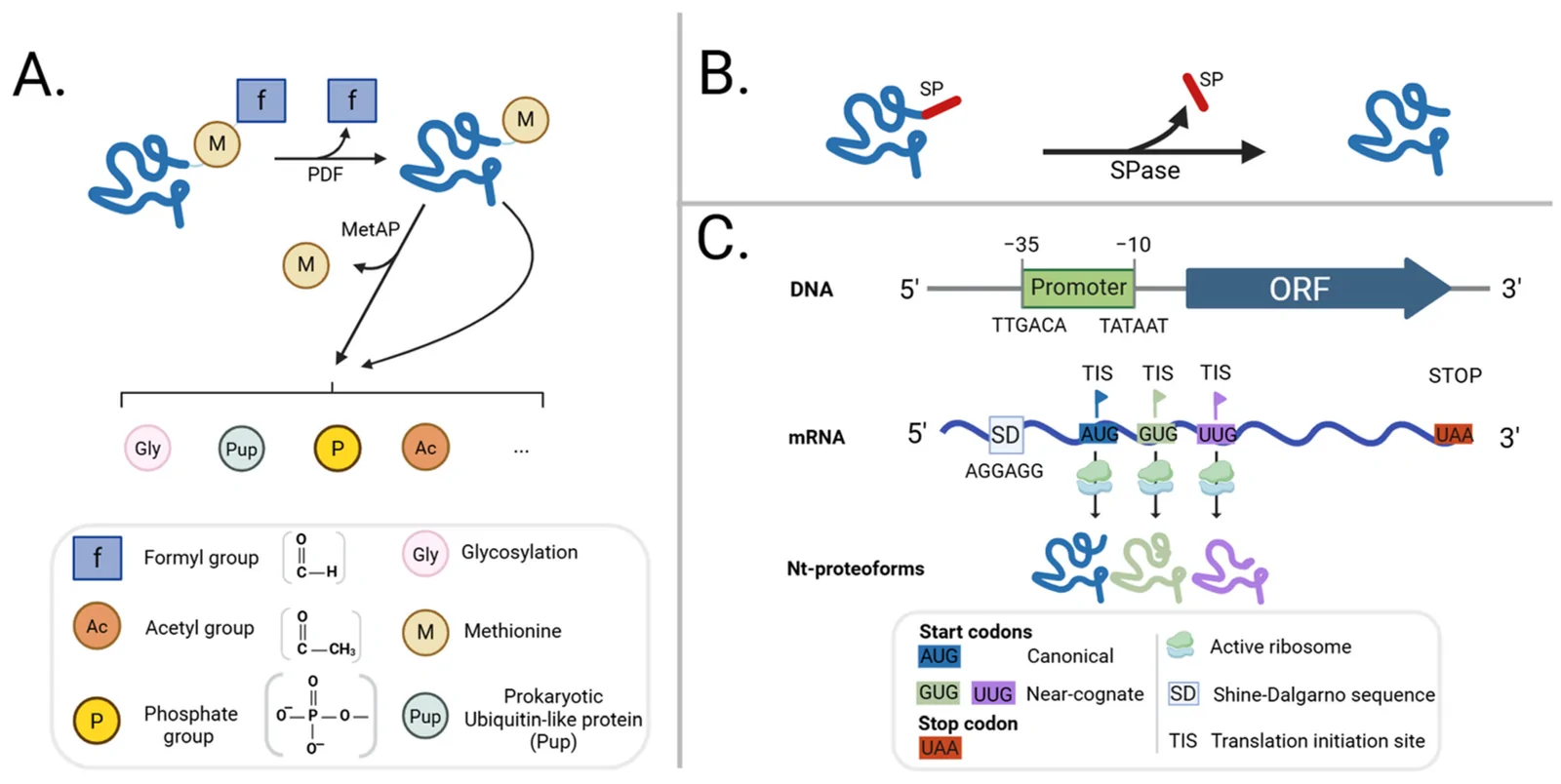
Mechanisms contributing to bacterial N-terminal (Nt-) proteoform diversity. Distinct Nt-proteoforms can arise through multiple co- and post-translational mechanisms that collectively expand bacterial proteome complexity. (**A**) **N-terminal processing and protein modifications**: Nascent bacterial proteins initially carry a formylmethionine residue. The Nt-formyl group (f) can be removed co-translationally by peptide deformylase (PDF), after which the initiator methionine (M) may either be retained or cleaved by methionine aminopeptidase (MetAP). Mature proteins may subsequently undergo additional post-translational modifications (PTMs), including phosphorylation, pupylation (addition of a prokaryotic ubiquitin-like protein), glycosylation, and other modifications, thereby further contributing to N-terminal heterogeneity. The “…” indicates that additional PTMs beyond those explicitly depicted may occur. (**B**) **Proteolytic processing**: Cleavage of N-terminal signal peptides (SPs) by dedicated signal peptidases (SPases) generates mature processed proteoforms with altered N-termini following protein translocation. (**C**) **Alternative translation initiation**: Multiple proteoforms can originate from a single gene through the use of alternative translation initiation sites (aTISs) within the same transcript. Translation may initiate from the canonical AUG start codon or from near-cognate initiation codons such as GUG and UUG. Promoter architecture, including the −10 and −35 promoter elements, as well as the Shine–Dalgarno (SD) sequence involved in ribosome binding and recruitment, is indicated. Utilization of distinct TISs results in Nt-proteoforms differing in N-terminal composition and length. Created in BioRender. Simoens, L. (2026) https://BioRender.com/ob8jk13.

In bacteria, proteome complexity is substantially amplified by alternative translation initiation events [3,4,5] and a broad repertoire of co- and post-translational modifications [6], collectively generating a diverse array of proteoforms (Figure 1). Proteoforms encompass all distinct protein variants derived from a single genetic locus [7,8].

Among bacterial protein modifications, one of the most prevalent is the co-translational removal of the N-terminal formyl group from the initiator methionine (iMet) by peptide deformylase (PDF), which associates with the ribosomal exit tunnel during translation elongation [9,10,11,12]. This processing event affects more than 94% of proteins in *Escherichia coli* (*E. coli*) [13] and exposes a free N-terminal amine group that permits further N-terminal maturation. Subsequently, methionine aminopeptidase (MetAP) may remove the iMet depending on the identity of the penultimate amino acid residue, a process estimated to affect more than half of mature bacterial N-termini [13,14,15].

In addition, protein N-termini may undergo (partial) Nt-acetylation mediated by N-terminal acetyltransferases (NATs), a process that occurs post-translationally in bacteria [16]. NATs can irreversibly transfer an acetyl group from acetyl-coenzyme A (acetyl-CoA) to the α-amino group of either the deformylated initiator methionine or the newly exposed N-terminal residue following iMet excision. Although bacterial N-terminal acetylation is generally observed at substoichiometric levels, up to 10–15% of bacterial proteins are estimated to contain detectable N-terminal acetylation [17,18,19,20,21]. Other protein modifications, including phosphorylation, lipidation, glycosylation, and pupylation, further contribute to bacterial proteome diversification (Figure 1A). Proteoform diversity is additionally expanded through coordinated proteolytic processing events, most notably the cleavage of signal peptides by dedicated signal peptidases [22]. Prior to removal, these N-terminal sequences serve essential regulatory functions by directing subcellular localization and mediating interactions with molecular chaperones that maintain proteins in a translocation-competent state. Following signal peptide cleavage, the newly exposed N-terminal residue may undergo further maturation. For example, in bacterial lipoproteins, cleavage exposes a conserved N-terminal cysteine that is subsequently lipidated, thereby promoting membrane anchoring of the mature protein [23,24,25]. Together, these processing and maturation events ultimately generate the mature and correctly localized proteoform (Figure 1B) [26].

Among these protein variants, distinct molecular forms originating from the same gene locus represent a major source of bacterial protein diversity. Beyond the previously discussed N-terminal maturation and processing events, Nt-proteoforms may additionally arise through the selection of alternative translation initiation sites (aTISs) distinct from the database-annotated or canonical translation initiation sites (dbTISs) (Figure 1C). Translation from internal or external in-frame and out-of-frame start sites significantly expands the bacterial proteome and can yield Nt-proteoforms that differ in their N-terminal composition and, consequently, their biological properties [4,27]. These alternative initiation events generate functionally distinct proteins that contribute to a more complex and versatile “alternative” proteome than previously recognized.

Multiple protein variants originating from a single genetic locus can arise through a combination of transcriptional and translational mechanisms. At the transcriptional level, the use of alternative transcription start sites (TSSs) can directly influence the selection and usage of aTISs. For instance, TSSs located within annotated coding regions may generate shorter transcripts that alter the set of translation initiation sites accessible to the ribosome. Accordingly, the observation that some experimentally mapped TSSs reside precisely between dbTISs and aTISs further illustrates how transcriptional boundaries can shape alternative translation initiation patterns [4].

Alongside transcription, multiple translational features further influence TIS selection. Due to bacterial transcription–translation coupling, local mRNA secondary structures or riboswitches may promote or inhibit translation initiation at specific TISs by masking or exposing ribosome binding sites (RBSs) [28,29,30,31,32,33,34]. In addition, the presence and strength of the Shine–Dalgarno (SD) sequences, together with their spacing relative to the initiating codon, strongly affect translation initiation efficiency. However, the presence of an SD sequence followed by an AUG triplet does not necessarily guarantee translation initiation [35], and because some leaderless mRNAs can be efficiently translated in the absence of an SD sequence [36], TIS selection remains highly dynamic and context-dependent.

Although AUG is the predominant start codon in *E. coli*, accounting for approximately 83% of annotated genes, near-cognate initiating codons such as GUG (14%) and UUG (3%) are also frequently utilized [37,38]. Similar patterns of start codon frequency have been observed across the bacterial domain [39]. Beyond these three major initiation codons, several additional non-AUG codons have been reported to support translation initiation [40]. Importantly, our previous work demonstrated that near-cognate start codons account for approximately one-quarter of newly identified Nt-proteoforms in *Salmonella enterica* serovar Typhimurium (*S.* Typhimurium) [5].

Collectively, these observations indicate that the bacterial translatome is not merely a static reflection of genome annotation, but rather a highly dynamic and adaptable landscape. The widespread occurrence of alternative translation initiation suggests that bacterial coding capacity extends substantially beyond canonical gene models, enabling the generation of multiple Nt-proteoforms from a single genetic locus [3,4,5]. These observations support the hypothesis that alternative translation initiation represents a biologically meaningful post-transcriptional regulatory mechanism rather than merely a source of stochastic translational events. Throughout this review, we examine the current evidence supporting this concept by discussing the biological relevance of Nt-proteoforms, recent advances in their riboproteogenomic discovery, and emerging genome-engineering approaches for their functional characterization.

## 2. Biological Relevance of Nt-Proteoforms

**Figure 2:**
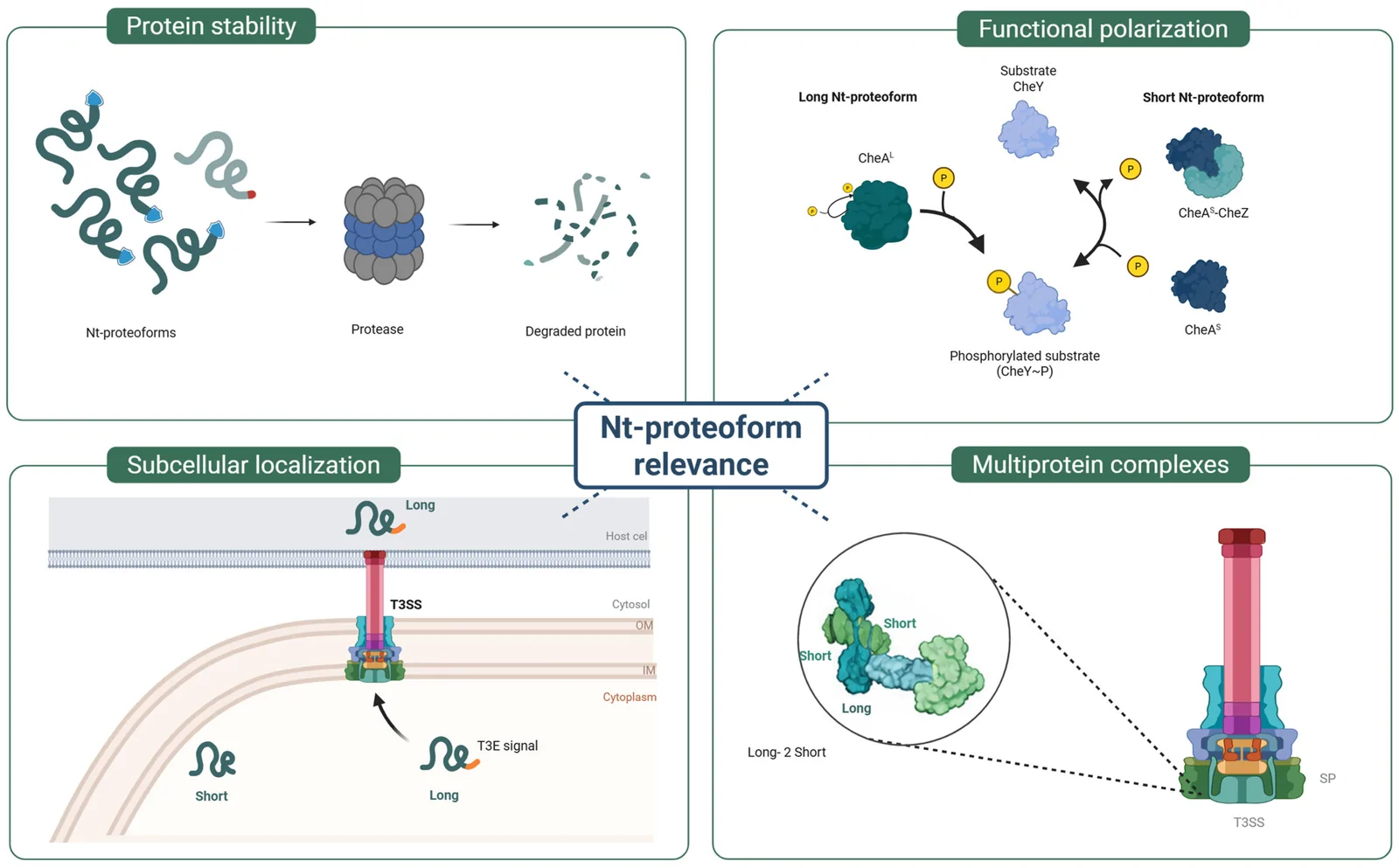
Biological relevance and functional implications of bacterial N-terminal (Nt-)proteoforms. The bacterial translatome constitutes a highly dynamic landscape in which N-terminal heterogeneity introduces multiple regulatory layers that influence protein fate and function. **Protein stability**: Variations at the N-terminus can modulate protein half-life through the bacterial N-degron pathway, in which specific N-terminal residues determine recognition by the ClpS-ClpAP proteolytic machinery. **Functional polarization**: Distinct N-termini can alter biochemical activity and signaling behaviour. The long CheA Nt-proteoform (CheA^L^) contains an N-terminal autophosphorylation site that is absent in the shorter CheA^S^ proteoform. Consequently, CheA^L^ primarily functions as a kinase that phosphorylates CheY, whereas CheA^S^ interacts with CheZ to promote CheY dephosphorylation. **Subcellular localization:** N-terminal extensions can encode transport or secretion signals, such as N-terminal type III effector (T3E) secretion signals, thereby enabling selective translocation of specific proteoforms (e.g., SseL^L^) into the host cell, while truncated proteoforms lacking these signals (e.g., SseL^S^) remain confined to the bacterial cytoplasm. **Multiprotein complex assembly**: Nt-proteoform diversity can further regulate the stoichiometric assembly and functionality of higher-order molecular complexes, exemplified by the T3SS sorting platform (SP), where defined ratios between long and short isoforms (e.g., one SpaO^L^ and two SpaO^S^ subunits) are required for proper injectisome assembly and activity. Created in BioRender. Simoens, L. (2026) https://BioRender.com/1s3coqt.

Heterogeneity at the N-terminus of bacterial proteins represents an important regulatory layer that may influence subcellular localization, multiprotein complex assembly, protein stability, and ultimately protein function (Figure 2).

To validate the hypothesis that alternative translation initiation represents a biologically meaningful regulatory mechanism rather than random translational variation, candidate proteoform pairs should exhibit distinct spatial, structural, or functional properties. Emerging case studies across diverse bacterial species provide compelling evidence supporting this hypothesis. N-terminal variation fundamentally dictates spatial organization, as illustrated by the architecture of cyanobacterial *β*-carboxysomes. Within these proteinaceous microcompartments, ribulose-1,5-biphosphate carboxylase/oxygenase (Rubisco), the key enzyme of the Calvin cycle, is sequestered to optimize CO_2_ fixation efficiency [41]. In *Synechococcus* sp. strain PCC 7942, an internal GUG_216_ initiation codon within *ccmM* generates two distinct isoforms, forming an N-terminal proteoform pair consisting of the full-length CcmM58 (58 kDa) and the N-terminally truncated CcmM35 (35 kDa). This 23 kDa difference fundamentally dictates their cellular functions. CcmM58, retaining its complete N-terminal region, is localized to the inner shell of the carboxysome, where it recruits the carboxysomal carbonic anhydrase CcaA and interconnects outer shell components [42,43,44]. Conversely, the truncated CcmM35 proteoform is confined to the carboxysome lumen and functions exclusively in organizing Rubisco into a paracrystalline matrix. This example highlights how N-terminal diversity can physically segregate structural and scaffolding functions within a single microcompartment to ensure efficient CO_2_ fixation.

Beyond mediating spatial organization within intracellular bacterial microcompartments, Nt-proteoforms can additionally control protein targeting and secretion in bacterial virulence systems. In bacterial pathogens such as *S*. Typhimurium, more than 40 type III effector (T3E) proteins are secreted through the type III secretion system (T3SS) to manipulate host cell biology and promote bacterial survival, replication, and dissemination [45,46,47]. The effector SseL exists as two proteoforms: a long isoform (SseL^L^, 38 kDa) and a shorter isoform lacking 23 N-terminal residues (SseL^S^, 29 kDa) [5]. The N-terminal extension unique to SseL^L^ contains a T3E secretion signal, thereby enabling translocation into the host cell environment. In contrast, SseL^S^ remains confined to the bacterial cytosol and may therefore fulfill distinct intracellular functions. In this context, it is important to note that T3Es were long assumed to remain inactive within the bacterium and only adopt their functional conformations following host-cell translocation, owing to chaperone-mediated maintenance of a secretion-competent unfolded state. However, recent studies have demonstrated that certain T3Es can exert important intrabacterial activities involved in the regulation of bacterial physiology [48,49]. This heteromorphic phenomenon is further corroborated by CyaA’ (calmodulin-dependent adenylate cyclase) translocation assays demonstrating that the SseL leader sequence constitutes the minimal determinant required for host-cell entry [50]. Accordingly, several additional candidate Nt-proteoform pairs, including SsaQ, YdgA, YfhG, MgtC, OmpX, and PagC, are predicted to exhibit distinct subcellular localization among their constituting Nt-proteoform members in *Salmonella* [5].

Beyond influencing effector localization and host-cell targeting, Nt-proteoforms can additionally modulate the assembly and stoichiometry of larger multiprotein machineries such as the T3SS sorting platform. The T3SS injectisome itself is composed of a cytosolic sorting complex, multiple membrane-embedded structures, a hollow needle spanning the bacterial membranes, and a translocon complex located at the tip (extensively reviewed in [51,52]). Central to this machinery is the cytosolic sorting platform, a multiprotein complex positioned at the base of the injectisome that coordinates effector sorting and unfolding prior to secretion. Previous studies demonstrated that SpaO^S^ (11 kDa) forms homodimers that subsequently associate with a single SpaO^L^ (34 kDa) subunit to generate a heterotrimeric complex with a strict 2:1 stoichiometry (2 SpaO^S^:1 SpaO^L^). Within this assembly, SpaO^L^ acts as a central interaction hub that promotes SP pod formation [53,54]. Although deletion of SpaO^S^ partially impairs T3SS functionality, secretion activity is not completely abolished, suggesting an ancillary yet functionally important role in T3SS-mediated protein translocation [54,55]. Consistent with this observation, deletion of the homologues Spa33^S^ (12 kDa) (a proteoform in *Shigella*) resulted in residual, albeit detectable, T3SS activity. Conversely, conventional in vitro studies in both *Yersinia* [56,57] and *Shigella* [58] indicate that the short SpaO^S^ isoform is indispensable for proper T3SS function.

In addition to controlling protein localization and multiprotein complex assembly, Nt-proteoforms may also directly modulate biochemical activity and signaling behaviour, as exemplified by the chemotaxis regulator CheA. This histidine kinase functions as a central regulator of bacterial chemotaxis. Upon binding of chemical stimuli to chemoreceptors, CheA undergoes autophosphorylation, thereby initiating a signal transduction cascade that governs flagellar rotation, receptor adaptation, and methylation [59,60]. In *E. coli*, an internal AUG_98_ initiation site generates the shorter CheA^S^ (60 kDa) isoform alongside the full-length CheA^L^ (71 kDa) isoform. The N-terminal difference between these variants dictates both their complex assembly and biochemical activities. CheA^L^ forms both homodimers and heterodimers with CheA^S^ and functions exclusively as a kinase responsible for phosphorylating the response regulator CheY [61]. In contrast, CheA^S^ lacks the critical His_48_ residue and instead fulfils distinct regulatory functions. Specifically, CheA^S^ localizes as small foci at the cell poles and participates in both phosphorylating and dephosphorylating complexes [62,63]. Furthermore, CheA^S^ interacts with and enhances the activity of CheZ-mediated dephosphorylation of phospho-CheY, while CheA^L^ lacks this capability [63,64]. This example illustrates how N-terminal heterogeneity enables a single genetic locus to generate proteins with functionally polarized roles.

Besides influencing localization, multiprotein complex assembly, and biochemical specialization, Nt-proteoforms may also profoundly affect protein stability. Importantly, not all Nt-proteoforms differ by extensive N-terminal truncations or extensions, as many identified proteoform architectures vary by only a few amino acid residues [4,5]. Nevertheless, even these subtle N-terminal differences may drastically influence protein stability through the N-degron pathway (formerly the N-end rule pathway), in which the identity of the N-terminal residue acts as a primary determinant of proteolytic fate [65,66,67]. In bacteria, this pathway is primarily mediated by the ATP-dependent ClpAP and its adaptor ClpS, a 12-kDa Leu/N-recognin that captures substrates and delivers them to the protease machinery [68]. ClpS preferentially recognizes primary destabilizing residues such as Leu, Phe, Trp, and Tyr, whereas proteins carrying stabilizing N-terminal residues (Met, Ser, Thr, Ala, Val, and Gly) generally exhibit prolonged half-lives. Although bacterial N-degron pathways firmly establish the importance of N-terminal identity in determining protein stability, endogenous Nt-proteoform architectures differing only minimally at their N-termini have rarely been directly linked to divergent degradation kinetics. However, analogous functional consequences of subtle N-terminal variation are well established in eukaryotes, where human Nt-proteoforms differing by only a few residues can display markedly distinct stability profiles [69].

## 3. Mapping the Bacterial Translatome: Genome Annotation, Ribosome Profiling, and N-Terminomics

**Figure 3:**
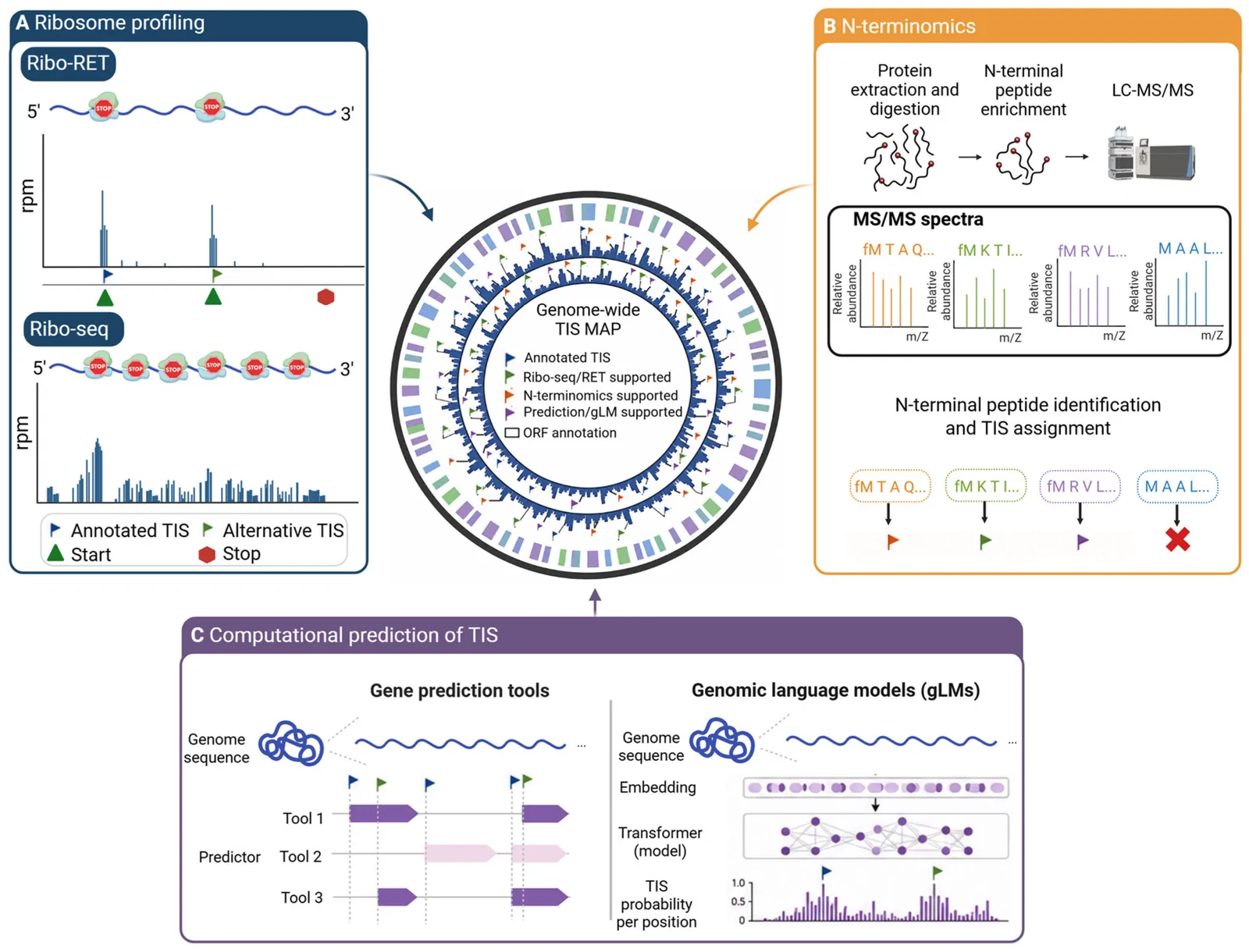
Integrated riboproteogenomic framework for high-resolution translation initiation site (TIS) mapping and bacterial genome annotation. Complementary experimental and computational approaches collectively enable the identification and validation of annotated and alternative translation initiation sites (aTISs) within bacterial genomes. The central circular overview integrates genome-wide TIS mapping results supported by ribosome profiling, N-terminomics, and computational prediction approaches. Annotated TISs, experimentally supported aTISs, and computationally predicted TISs are represented alongside the corresponding open reading frame (ORF) architecture. The “…” indicates that the protein sequence continues beyond the (four) N-terminal amino acids depicted. (**A**) Ribosome profiling. Conventional Ribo-seq captures actively translated genomic elements through ribosome-protected mRNA fragments, with expression levels quantified as reads per million (RPM). Specialized approaches such as retapamulin-assisted ribosome (Ribo-RET) enrich initiating ribosomes through antibiotic-mediated stalling at start codons, thereby improving the detection of canonical and aTISs. (**B**) N-terminomics. N-terminal proteomics workflows combine protein extraction, proteolytic digestion, selective enrichment of N-terminal peptides, and LC-MS/MS analysis to identify proteoform-specific N-termini. The presence of formylated methionine (fM) serves as a direct proxy for an actual initiator methionine. Consequently, detection of fM-containing N-terminal peptides provides direct molecular evidence for translation initiation events and enables TIS delineation at single-amino-acid resolution. (**C**) Computational TIS prediction. Computational approaches ranging from classical ab initio gene finders (e.g., GeneMark, Prodigal, and Glimmer) to modern genomic language models (gLMs) are used to predict coding sequences (CDSs) and TISs. Created in BioRender. Simoens, L. (2026) https://BioRender.com/yiegp9n.

The increasing prevalence of Nt-proteoforms within the bacterial translatome reveals a regulatory layer that is frequently overlooked by traditional genome annotation approaches. Accurate delineation of this proteomic landscape increasingly relies on integrated frameworks combining computational prediction with high-resolution experimental data (Figure 3). Three complementary pillars currently underpin this strategy: ribosome profiling (Figure 3A), which identifies actively translated regions and TISs; N-terminomics (Figure 3B), which provides direct mass spectrometry (MS-based) evidence of protein start sites; and computational modeling (Figure 3C), ranging from classical ab initio gene prediction algorithms to modern genomic language models (gLMs). Together, these complementary approaches are shifting bacterial genome annotation from static gene predictions toward dynamic translatome- and proteome-aware annotation strategies [70].

### 3.1. Classical Genome Annotation Approaches and Their Limitations

Historically, the existence of Nt-proteoforms became apparent through observations that single gene loci could produce multiple protein variants differing at their N-termini. However, accurately defining the genomic boundaries and translation initiation events underlying this heterogeneity remained technically challenging. At the turn of the millennium, the first generation of microbial genome annotation tools emerged, most notably Gene Locator and Interpolated Markov ModelER (GLIMMER) [71] and GeneMark [72]. These algorithms used probabilistic sequence models to identify coding regions within microbial genomes and represented major advances in automated gene prediction. However, early annotation tools were often computationally intensive and displayed limited accuracy in identifying (alternative) translation initiation sites (a)TISs. Consequently, the identification of bacterial Nt-proteoforms relied largely on isolated, serendipitous biochemical observations rather than systematic, genome-wide detection. As evidence accumulated that alternative translation initiation contributes to bacterial proteome complexity, there arose a need to transition from incidental discoveries towards integrated, translatome-wide approaches capable of systematically identifying translation initiation events.

Methodological development subsequently focused on improving the precision of TIS identification, most notably through the development of the PROkaryotic Dynamic programming Gene-finding Algorithm (Prodigal) [73]. Despite their widespread use, substantial discrepancies persist among annotation platforms. Comparative analysis of GLIMMER, GeneMark, and Prodigal revealed only 70% consensus between predictions, highlighting the inherent algorithmic biases and limitations within individual tools [74]. To meet increasing high-throughput demands, automated annotation pipelines such as the Institute for Genomic Research (TIGR) Annotation Engine [75] and the Bacterial Annotation System (BASys) [76] were subsequently developed to streamline these workflows. This evolution ultimately culminated in fully automated annotation services for archaeal and bacterial genomes, including Rapid Annotations using Subsystems Technology (RAST) [77], which was later integrated into the Bacterial and Viral Bioinformatics Resource Center (BV-BRC) [78]. Despite the comparatively simple gene architecture of prokaryotes relative to eukaryotes, modern annotation tools still struggle to resolve internal genomic complexity. Most annotation pipelines fail to detect multiple internal TISs (iTISs) within a single coding sequence (CDS). Widely used annotation frameworks such as the NCBI Prokaryotic Genome Annotation Pipeline (PGAP) rely heavily on homology-based methodologies, integrating Hidden Markov Models (HMMs), BlastRules, and curated Conserved Domain Database (CDD) architectures [79,80]. Currently, approximately 79% of the RefSeq proteins are annotated based on matches to a curated protein family model (PFM) [81]. Importantly, these tools frequently prioritize the longest possible ORF to maximize coding information, although this does not necessarily reflect the true bacterial translatome [70].

Although current ab initio gene prediction algorithms robustly identify canonical ORFs, their sensitivity declines substantially for non-canonical genomic elements. First, many annotation tools exclude sequences shorter than 150–300 nucleotides, thereby systematically overlooking small open reading frames (sORFs) [82,83,84]. Increasing evidence demonstrates that these small proteins participate in critical biological processes, including protein recruitment [85,86], protein stability [87,88], and multiprotein complex assembly [89,90,91], among others. Second, current annotation frameworks generally fail to identify aTISs that give rise to Nt-proteoforms. Homology-driven pipelines such as PGAP frequently enforce previously annotated start sites across related genomes, thereby masking biologically relevant variation in translation initiation [80]. Finally, annotation tools optimized for computational speed, such as RAST and Prokka (Prokaryotic Annotation), often show reduced performance when annotating specialized or highly divergent genes that lack closely related homologs.

### 3.2. Machine Learning and Genomics Language Models

Recent advances in machine learning are reshaping genome annotation strategies. In particular, gLMs, such as DNA-language Bidirectional Encoder Representations from Transformers (DNABERT) and Evo 2, interpret DNA sequences as structured sequential information, enabling the extraction of both local sequence motifs and long-range contextual relationships [92,93,94]. Compared with traditional alignment- or homology-based approaches, these models offer improved adaptability for resolving complex bacterial gene architectures, offering a more adaptive alternative to traditional genome annotation.

Various deep learning-based frameworks have been developed for bacterial gene prediction, including conventional neural networks [95]. More recently, gene language model (GeneLM), a DNABERT-based framework, was introduced for prokaryotic gene prediction using a two-stage approach involving initial CDS identification followed by precise TIS prediction across diverse bacterial species [96]. Benchmarking studies demonstrated that GeneLM consistently outperformed established gene prediction tools, including Prodigal, GeneMark, and GLIMMER, as well as specialized deep learning approaches for TIS prediction, such as Translation Initiation Site Detector (TITER) [97] and DeepGSR (Deep-learning Structure for the Recognition of Genomic Signals and Regions) [98], both of which exhibit comparatively lower TIS prediction performance.

Despite these advances, the performance of gLMs remains highly dependent on the quality and diversity of their training datasets. Nevertheless, gLMs are expected to provide increasingly accurate frameworks for identifying novel gene structures and refining gene boundaries in both well-characterized and previously unannotated genomes.

### 3.3. Ribosome Profiling-Based Translation Initiation Site Mapping

The limitations of purely computational annotation strategies underscore the importance of experimental validation and evidence-based annotation approaches. Proteogenomic (re-)annotation efforts have therefore been widely applied to identify novel genes and to correct missing or inaccurate annotations [99,100,101,102]. In particular, the rapid evolution of riboproteogenomics has largely been driven by the advent of ribosome profiling (Ribo-seq). General Ribo-seq approaches infer translation by capturing ribosome footprints across the transcriptome, thereby substantially improving ORF detection and Nt-proteoform discovery. One of the first bacterial genome annotation approaches integrating Ribo-seq-derived translational evidence was RibosomE Profiling Assisted (Re-)AnnotaTION (REPARATION), which enabled de novo ORF delineation in prokaryotic genomes [103]. Consequently, extensive re-annotation efforts have been performed in so-called well-annotated bacteria, including *E. coli*, *S.* Typhimurium, and *Bacillus subtilis* [82,103,104].

To move beyond general ORF delineation and improve TIS resolution, several specialized Ribo-seq approaches have been developed to selectively enrich initiating ribosomes. One of the earliest approaches, tetracycline-inhibited ribosome profiling (TetRP), was introduced as a relatively simple and comprehensive strategy for bacterial TIS annotation [105]. Tetracycline blocks aminoacyl-tRNA entry into the ribosomal A-site and is therefore classically regarded as an elongation inhibitor. To further improve TIS resolution, Meydan et al. [4] subsequently developed retapamulin-assisted ribosome profiling (Ribo-RET), which selectively enriches initiating ribosomes through retapamulin-mediated stalling. Using this approach, internal start codons were identified in more than one hundred *E. coli* genes. Application of Ribo-RET in *S.* Typhimurium further revealed 26 candidate Nt-proteoform pairs following manual curation [5], demonstrating that, in both *E. coli* and *S*. Typhimurium, the majority of identified aTISs cluster near the beginning of the CDSs.

Despite these methodological advances, precise TIS delineation remains highly challenging due to several technical and biological limitations. One major limitation stems from incomplete elongation inhibition during antibiotic treatment. Because tetracycline can interact with ribosomes throughout multiple elongation cycles, TetRP cannot reliably discriminate between elongating and initiating ribosomes, thereby limiting its sensitivity for detecting aTISs [105]. Retapamulin-based approaches face a related constraint, as signals originating from elongating ribosomes are not completely eliminated. This residual background complicates confident identification of internal start codons, particularly within highly expressed ORFs [106]. Furthermore, Ribo-RET peaks at putative initiation sites do not inherently demonstrate productive, full-length translation in the absence of antibiotic treatment, meaning that some detected events may represent abortive or non-functional initiation [107].

In addition to antibiotic-related artifacts, ribosome profiling approaches also suffer from intrinsic resolution constraints because translation initiation is inferred indirectly through ribosome footprints. Since translating ribosomes physically protect RNA fragments of approximately 22–35 nucleotides, overlapping footprints complicate precise discrimination between closely positioned TISs [108].

Moreover, the reliance of ribosome profiling on living cells can complicate the distinction between direct and indirect translational effects. Translation-targeting antibiotics and their associated uptake mechanisms may inadvertently induce generalized stress responses and widespread off-target transcriptional and translational changes that obscure native cellular physiology [109,110].

To minimize such confounding in vivo artifacts, in vitro Ribo-seq (INRI-seq) was developed as a cell-free alternative that enables translation analysis on customizable synthetic transcriptomes [111]. Although INRI-seq successfully confirmed the annotated TIS for approximately 70% of *E. coli* genes and validated 51 out of the 64 aTISs previously identified by in vivo Ribo-RET [4], the method remains constrained by its current library design, which captures only the first 50 codons of a CDS. Consequently, alternative TIS detection is inherently restricted to the first ~150 nucleotides of a gene, thereby limiting the identification of more downstream internal initiation events.

### 3.4. N-Terminomics Approaches for Nt-Proteoform Discovery

In conventional bottom-up proteomics workflows, alternative Nt-proteoforms are often difficult to distinguish from their canonical counterparts because most internal tryptic peptides are shared [112,113]. Proteoform-specific information is mainly confined to the altered N-terminal peptide and, in some cases, to the absence of peptides from the truncated N-terminal region, making dedicated N-terminal proteomics (N-terminomics) strategies essential for direct TIS identification. Together with ribosome profiling, N-terminomics has become an important tool for high-resolution identification of prokaryotic translation initiation sites (TISs).

Techniques such as Combined FRActional Diagonal Chromatography (COFRADIC) [114,115], Terminal Amine Isotopic Labeling of Substrates (TAILS) [116], and LysN Amino Terminal Enrichment (LATE) [117] employ selective chemical derivatization and chromatographic enrichment to isolate N-terminal peptides prior to LC-MS/MS analysis. By reducing sample complexity and enriching proteoform-specific N-terminal peptides, these approaches facilitate direct detection of both canonical and alternative TISs. Comprehensive overviews of bacterial N-terminomics strategies have previously been provided by Berry et al. [113]. Recently, a deformylation-assisted N-terminomics workflow termed TRAnslation Initiation SPOTTER (TRAINSPOTTER) was developed [118]. By exploiting the presence of the nascent N-formyl group—optionally stabilized through peptide deformylase (PDF) inhibition—TRAINSPOTTER enables proteome-wide detection of nascent N-termini and thereby provides direct molecular evidence for translation initiation at single-amino-acid resolution. Nevertheless, the efficacy of MS-based TIS delineation remains highly dependent on both the completeness of the underlying protein sequence databases and the applied database search strategy. The composition of the proteomic search space fundamentally defines the boundaries of novel proteoform discovery. Historically, unannotated TISs, including aTISs and initiation events originating from non-canonical start codons, frequently escaped MS-based detection because they were absent from standard reference databases. However, this limitation is not absolute and becomes particularly problematic when stringent enzymatic cleavage constraints are imposed during database searching or when non-AUG initiation events are excluded from the databases.

To circumvent these limitations, the TRAINSPOTTER workflow interrogates MS datasets against a customized six-frame translation (6-FT) database combined with semi-specific search parameters that account for iMet processing rules. This relatively unconstrained search strategy enables identification of internal and alternative N-termini, thereby providing direct physical evidence for productive translation initiation from both canonical and non-canonical start codons.

Despite these advances, current N-terminomics workflows remain limited in their ability to capture TIS-indicative N-termini of proteins undergoing signal peptide cleavage. In such cases, the original N-terminus is removed during protein translocation, effectively erasing the molecular evidence of the initial TIS and complicating comprehensive mapping of the bacterial N-terminal proteome landscape.

## 4. Methodological Strategies for Decoupling Bacterial N-Terminal Proteoforms

While the growing number of identified aTISs reveals a highly modular bacterial proteome, the capacity for high-throughput discovery currently exceeds downstream functional validation. Consequently, substantial gaps remain in our understanding of the precise physiological roles of individual Nt-proteoforms.

### 4.1. Classical Heterologous Complementation Approaches

Initial functional characterization and decoupling of bacterial Nt-proteoforms—defined as the selective expression and analysis of individual proteoforms—primarily relied on deletion of the endogenous gene followed by heterologous complementation. Within this framework, individual proteoforms are typically expressed from (inducible) plasmids to evaluate their ability to complement a mutant phenotype. This strategy provided some of the earliest functional insights into the isoforms of Initiation Factor 2 (IF2), encoded by *infB*. Expression of IF2α and IF2β from pEV1 and pBAD24 vectors enabled characterization of their distinct ribosomal binding properties and respective contributions to replication restart [119,120]. Similarly, plasmid-based complementation experiments demonstrated that a 46-amino-acid shorter proteoform of the major penicillin-binding protein PBP-1B, encoded by *mrcB*, could restore thermosensitive growth defects in *E. coli* [121]. Another classical example is represented by the ClpB proteoform pair, originally identified due to pronounced molecular weight differences and subsequently decoupled using pBS- or pGB2-based expression systems [122,123,124].

Despite their widespread utility in gain-of-function studies, heterologous expression systems frequently fail to recapitulate the native biological context. Plasmid-based protein overexpression often drives protein concentrations far beyond physiological levels, potentially inducing molecular crowding, aberrant subcellular localization, inclusion body (IB) formation, or artificial protein–protein interactions (PPIs). For example, overexpression of periplasmic proteins may saturate Sec-translocon capacity, thereby impairing the secretion of endogenous proteins and promoting cytoplasmic protein aggregation [125]. Similarly, excessive production of aggregation-prone proteins can promote artificial non-physiological PPIs or sequester essential factors into toxic aggregates [126]. Such artifacts are particularly disruptive for multiprotein complexes and signaling pathways, where strict stoichiometric balance is essential for proper function. Furthermore, proteoform expression is often tightly coupled to specific growth phases or environmental stimuli [122,127,128,129,130,131], whereas inducible plasmid systems frequently bypass the native transcriptional and post-transcriptional regulatory architecture. Finally, episomal maintenance often requires continuous antibiotic selection, imposing an additional metabolic burden that may fundamentally alter cellular physiology [132].

### 4.2. Endogenous Chromosomal Manipulation Strategies

To overcome the limitations associated with heterologous overexpression systems, increasing emphasis has been placed on endogenous genome engineering strategies. By manipulating genes directly within their native chromosomal context, proteoform expression remains under endogenous promoter and regulatory control. This approach can be combined with small high-sensitivity peptide tags, such as HiBiT or 3 × FLAG, to facilitate proteoform detection at physiological expression levels while avoiding disruptive overexpression [133,134].

The *S*. Typhimurium protein SpaO represents a landmark example of Nt-proteoform decoupling via endogenous genome manipulation. The *spaO* gene contains an internal GTG_203_ initiation codon to yield two distinct proteoforms: the full-length SpaO^L^ and the truncated SpaO^S^ proteoforms. To dissect their individual functions, Lara-Tejero et al. employed R6K-based allelic exchange to steer selective Nt-proteoform expression [54,135]. This suicide plasmid-based allelic exchange strategy enables precise chromosomal mutagenesis through homologous recombination. Following counterselection using markers such as *sacB*, a second recombination event removes the plasmid backbone and leaves behind the desired scarless mutation. Specifically, this approach was used to mutate the internal GTG_203_ start codon to the non-initiating GCG codon, successfully eliminating SpaO^S^ production and enabling selective functional interrogation of SpaO^L^. Additionally, loss-of-function (*∆spaO* mutant) phenotypes were complemented by expressing a wild-type *spaO* copy in trans from the pSB4545 plasmid.

### 4.3. (Multiplex) Recombineering Approaches

More recent developments have implemented multiplexed recombineering approaches to precisely manipulate Nt-proteoform expression while simultaneously enabling high-sensitivity detection through integration of peptide tags such as the luminescent HiBiT tag [5]. This 11-amino-acid peptide (VSGWRLFKKIS) associates with high affinity to the complementary Large BiT (LgBiT) subunit to form an active luciferase, thereby enabling highly sensitive bioluminescent quantification. A cornerstone of modern bacterial genome engineering is the λ-Red recombineering system, which mediates homologous recombination through the phage-derived Gam, Bet, and Exo proteins. This platform enables precise genomic engineering using either single-stranded DNA (ssDNA) oligonucleotides or dsDNA repair templates [136].

The use of ssDNA oligonucleotides, commonly referred to as oligo-mediated allelic replacement (OMAR) [137], is particularly well suited for Nt-proteoform decoupling because it permits seamless conversion of individual TIS codons into non-initiating codons without introducing selection markers that could perturb operon polarity. Maintaining operon integrity is biologically important in prokaryotes, where genes are often tightly clustered and co-transcribed. Residual recombination scars or selection markers may disrupt important transcriptional and translational regulatory sequences, including promoters, terminators, and RBSs [138,139,140]. Consequently, OMAR is particularly well suited for TIS mutagenesis while preserving the native physiological context of the targeted genetic locus.

Nevertheless, careful oligonucleotide design remains essential when modifying start codons. Near-cognate codons such as ATA should generally be avoided because they may still permit low-level “leaky” translation initiation [40,141]. Instead, codons lacking initiation potential are preferred, ideally while preserving the encoded amino acid or introducing only conservative amino acid substitutions where possible. For example, CTT can serve as a reliable non-initiating alternative in certain leucine-compatible contexts [40]. Furthermore, because closely spaced TISs may overlap with SD-like sequences or other cis-regulatory elements, even single nucleotide substitutions may inadvertently disrupt translational coupling or generate unintended polar effects across polycistronic operons [142,143,144]. Predictive tools such as the Genetic Systems Calculator [145] therefore provide valuable support for evaluating the translational consequences of engineered mutations.

Beyond design constraints, the execution of precise tag-free TIS modifications poses substantial screening challenges. Because OMAR typically generates scarless and non-selectable mutations, the identification of correctly edited clones becomes experimentally challenging. Reported baseline efficiencies for OMAR vary considerably, typically ranging from 6% to 20% depending on the genetic background, mutation type, and oligonucleotide modifications [137]. To overcome these low baseline frequencies in the absence of direct selection, co-selection MAGE (Cos-MAGE) can be implemented. By co-targeting a nearby selectable chromosomal marker with an additional ssDNA oligo, CoS-MAGE enriches for subpopulations containing both mutations, thereby substantially increasing the allelic replacement frequency (ARF) of the unselectable TIS mutation [146].

To identify successful mutants within these enriched populations, alternative screening approaches such as allele-specific colony PCR (ASC-PCR) are required [147]. While OMAR efficiencies have historically been estimated using selectable chromosomal markers that generate easily screenable phenotypes on selective media—thereby enabling ARF calculation as a percentage of the surviving population [148]—our previous work demonstrated that ASC-PCR enables the determination of actual TIS conversion frequencies [149]. Broader optimization strategies for recombineering efficiency have been comprehensively reviewed elsewhere [130].

Overall, multiplexed recombineering approaches have previously been shown to effectively steer Nt-proteoform expression at the genomic level while simultaneously allowing sensitive detection of endogenous expression [5]. Decoupling Nt-proteoform expression is particularly relevant for validation purposes when multiple TISs are located in close proximity within the bacterial genome. Furthermore, this approach enables characterization of conditionally expressed proteoforms, as exemplified by the *S*. Typhimurium FruK and *E. coli* RNA polymerase Sigma S (σ^S^) proteoform pairs [5,150].

### 4.4. CRISPR-Based Nt-Proteoform Engineering

Since the development of clustered regularly interspaced short palindromic repeats (CRISPR)-based genome engineering, CRISPR/Cas (CRISPR-associated) systems have become widely implemented for bacterial genome manipulation [151,152,153,154]. These approaches rely on guide RNA (gRNA)-directed Cas-mediated cleavage at target protospacer sequences adjacent to protospacer-adjacent motifs (PAMs), thereby enabling counterselection of nonedited wild-type cells. Importantly, CRISPR-based strategies can eliminate the need for selectable markers, classical counterselection systems, or dedicated recombineering platforms. Many early CRISPR/Cas-based bacterial editing strategies required the introduction of additional mutations within the protospacer or PAM sequence to prevent repeated Cas-mediated cleavage following successful genome editing. While initial CRISPR-Cas9-mediated oligonucleotide-directed mutagenesis approaches achieved relatively high editing efficiencies for two- to three-base mutations, introduction of single-nucleotide mutations proved substantially more challenging [155,156]. Such single-base edits were often obtained at frequencies below 3%, largely due to the mismatch tolerance of the CRISPR/Cas system [157]. However, subsequent optimization of sgRNA design strategies dramatically improved single-nucleotide editing efficiencies to between 36% and 95% [157,158].

More recently, the RECKLEEN platform combines λ-Red recombineering with CRISPR-Cas9-mediated counterselection, achieving editing efficiencies approaching 100% in *Klebsiella* [159]. Nevertheless, CRISPR-mediated genome engineering still faces several important limitations, including off-target mutagenesis and the occurrence of “escaper” colonies containing wild-type clones or unintended genomic alterations [160]. In addition, excessive Cas9 expression may exert cytotoxic effects, thereby limiting the broader applicability of certain CRISPR-based editing systems [160]. Despite these limitations, increasingly precise CRISPR-based genome engineering technologies hold potential for Nt-proteoform research by enabling selective manipulation of endogenous proteoform expression within native chromosomal contexts.

## 5. Functional Characterization of Nt-Proteoforms

The rapid expansion of computational TIS prediction, ribosome profiling, and N-terminomics datasets has substantially increased the number of candidate bacterial Nt-proteoforms [4,5,96,118]. Nevertheless, functional validation and mechanistic characterization of these proteoforms remain comparatively limited. Recent advances in endogenous genome engineering now enable the selective decoupling of individual proteoforms within their native chromosomal context, thereby facilitating direct investigation of their physiological relevance [149]. Such strategies provide opportunities to study proteoform-specific effects on cellular physiology, subcellular localization, interaction networks, stability, and conditional regulation.

**Figure 4:**
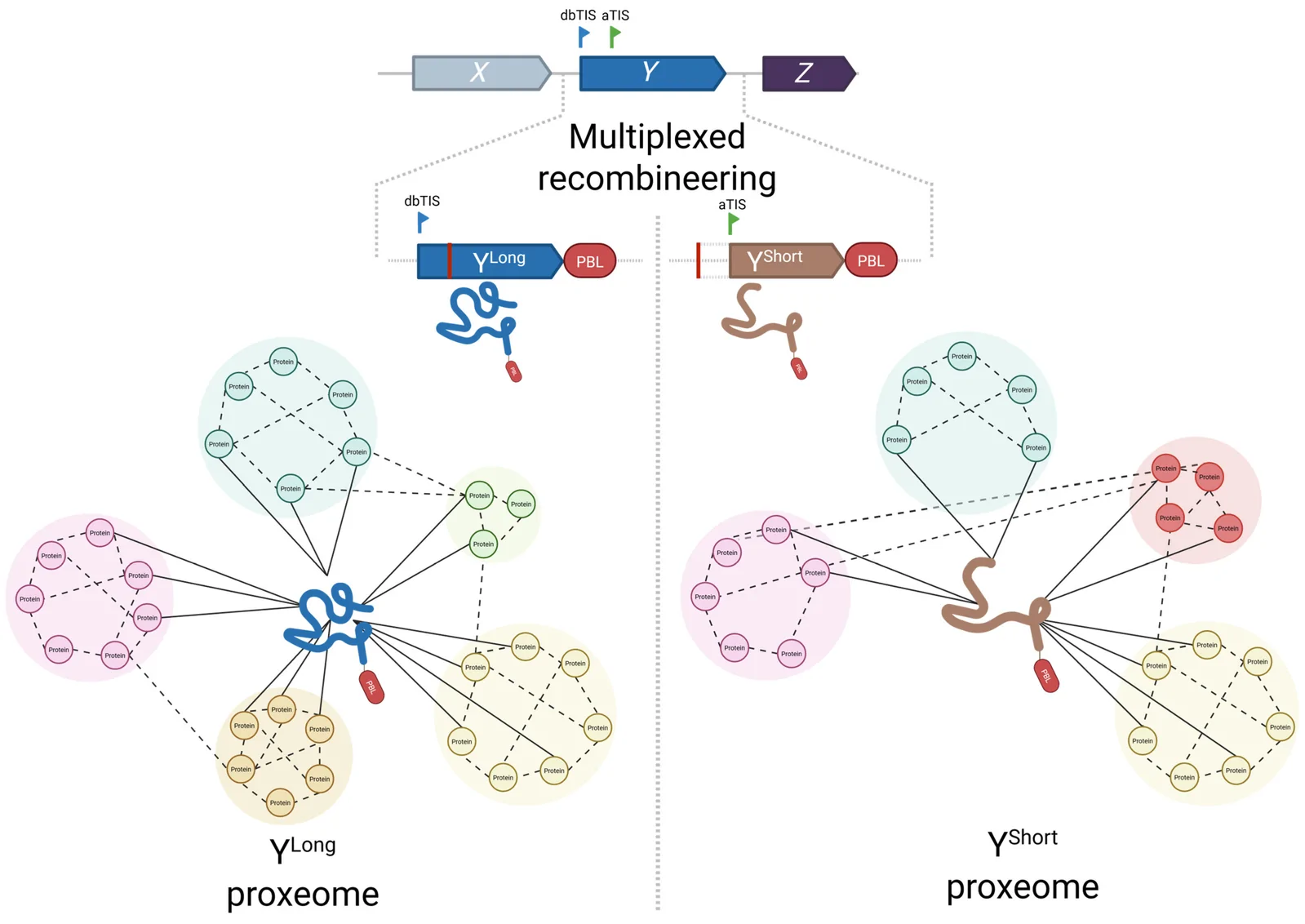
Multiplexed recombineering enables proteoform-specific proximity interactome (”proxeome”) mapping. Schematic representation of a bacterial genomic locus comprising genes X, Y and Z. Gene Y encodes two Nt-proteoforms generated through alternative translation initiation. The long proteoform (Y^Long^) is translated from the database-annotated translation initiation site (dbTIS; blue flag), whereas the short proteoform (Y^Short^) originates from an alternative translation initiation site (aTIS; green flag). Multiplexed recombineering is used to selectively inactivate either TIS while simultaneously integrating a C-terminal promiscuous biotin ligase (PBL) fusion. This genome engineering strategy generates strains selectively expressing either Y^Long^ or Y^Short^ -PBL under endogenous regulatory control. Subsequent proximity-dependent biotin identification (BioID) approaches enable mapping of the specific proxeomes associated with each Nt-proteoform. Solid lines denote direct or stable protein interactions, whereas dashed lines represent indirect, transient, or shared interaction networks. Created in BioRender. Simoens, L. (2026) https://BioRender.com/x9pzs2y.

Recently, multiplexed recombineering toolkits have been extended toward genomic integration of promiscuous biotin ligases (PBLs), thereby enabling proximity-dependent biotinylation approaches such as BioID [149]. These systems exploit engineered ligases, including BioID/BirA* [161], BioID2 [162], TurboID, and miniTurboID [163], to map local protein interaction environments, although their application in prokaryotes remains limited [164,165]. Resolving the specific proxeomes of decoupled Nt-proteoforms may provide important insights into how N-terminal variation influences protein interactions, complex assembly, and functional specialization (Figure 4) [166,167].

Nt-proteoform variation may additionally influence protein stability. In eukaryotes, altered N-termini have been shown to substantially affect proteoform half-life [69]. Protein stability is commonly investigated using pulse-chase approaches such as pulse SILAC (pSILAC) combined with COFRADIC, which enables temporal monitoring of proteoform degradation dynamics [115,118,168,169,170,171]. Although initially developed for eukaryotic cells, recent methodological adaptations have enabled highly efficient SILAC labeling in bacteria, thereby expanding the applicability of these approaches to prokaryotic proteome dynamics [172]. Interestingly, a recent *E. coli* study suggested that N-terminal identity may not always represent the dominant determinant of protein stability in vivo [173]. However, unstable proteoforms may evade detection entirely due to rapid turnover, complicating the interpretation of such datasets.

Collectively, the combination of precise endogenous genome engineering with complementary proteomic and interaction-mapping technologies provides an emerging framework for systematic functional characterization of bacterial Nt-proteoforms.

## 6. Discussion

Although Nt-proteoforms have now been identified across phylogenetically diverse bacterial species, their overall prevalence and evolutionary distribution across bacterial phyla remain largely unresolved. Current evidence is still largely limited to a relatively small number of experimentally investigated lineages, spanning taxonomically distant groups such as *Streptomyces* and *Cyanobacteria* [42,174]. However, recent riboproteogenomic advances are increasingly enabling more systematic and large-scale identification of bacterial Nt-proteoforms [4,5,118]. By revealing a far more dynamic and heterogeneous proteomic landscape than previously appreciated, these methods have fundamentally transformed our understanding of the bacterial translatome. In particular, the widespread occurrence of aTIS events demonstrates that single bacterial loci can generate multiple Nt-proteoforms with potentially distinct biological properties. However, the rapid expansion of high-throughput discovery datasets is currently outpacing downstream functional characterization, leaving the physiological relevance of many candidate Nt-proteoforms unresolved.

A central challenge in the field is distinguishing functional Nt-proteoforms from pervasive or non-productive translational noise [175,176]. Ribosome profiling approaches such as Ribo-RET provide highly sensitive snapshots of ribosome occupancy and initiation-site selection but fundamentally measure ribosome-protected mRNA fragments, rather than stable protein products [4]. Consequently, not every detected initiation event necessarily yields a biologically relevant proteoform. Nevertheless, our previous work demonstrated that aggregate Ribo-seq and Ribo-RET signals correlate strongly with experimentally determined protein abundances. This correlation indicates that ribosome occupancy metrics can serve as valuable quantitative proxies for translation output [5] and that signal intensity, reproducibility, positional conservation, sequence context, and integration with complementary proteomic evidence serve as critical parameters for prioritizing candidate functional Nt-proteoforms.

Accordingly, orthogonal peptide-level validation remains essential. N-terminomics approaches such as TRAINSPOTTER provide direct molecular evidence for productive translation initiation by experimentally identifying proteoform-specific N-termini [118]. The integration of quantitative ribosome profiling with high-confidence N-terminal proteomics, therefore, represents a particularly powerful framework for distinguishing genuine proteoforms from pervasive translational noise and for delineating bacterial translation initiation events at single-amino-acid resolution.

From an evolutionary perspective, alternative translation initiation may represent an efficient strategy for expanding bacterial functional capacity without increasing genome size. Bacterial genomes are highly compact and coding dense, with approximately 90% of genomic DNA dedicated to coding regions [177]. Within this constrained genomic architecture, the generation of multiple Nt-proteoforms from a single transcript provides a mechanism to diversify protein functionality while minimizing the genetic burden associated with gene duplication. In this regard, Nt-proteoform generation may represent an efficient strategy for expanding proteomic and regulatory complexity in streamlined prokaryotic systems.

Beyond coding efficiency, alternative translation initiation may additionally provide important regulatory and kinetic advantages. Because transcription and translation are tightly coupled in bacteria, modulation of translation initiation can enable highly rapid proteomic adaptation without requiring de novo transcriptional programs [178]. Changes in local mRNA structure, ribosome binding site accessibility, or environmental cues may rapidly shift TIS usage and thereby alter proteoform production under changing growth conditions. Such mechanisms could facilitate fast post-transcriptional remodelling of the bacterial proteome during environmental, thermal, nutritional, or host-associated stress responses. Consistent with this concept, several characterized proteoform-encoding genes, including *if2*, *clpB*, *safA*, and *σS*, display condition-dependent expression or specialized stress-associated functionality [129,179,180].

Alternative translation initiation may also contribute to stoichiometric regulation of multi-protein complexes. In the T3SS sorting platform of *S*. Typhimurium, defined ratios between long and short SsaQ and SpaO proteoforms are required for proper injectisome assembly and functionality [54,55,181]. Encoding multiple structural variants within a single transcriptional unit may therefore provide a robust mechanism to coordinate relative subunit abundance while minimizing the need for additional transcriptional regulatory layers.

In addition, Nt-proteoform diversity may influence protein stability and conditional protein turnover. Although bacterial N-degron pathways clearly establish the importance of N-terminal identity in proteolytic targeting, endogenous Nt-proteoforms differing only minimally at their N-termini have thus far rarely been systematically linked to distinct degradation kinetics. It nevertheless remains highly plausible that certain alternative proteoforms evolved as transient or condition-specific molecular variants with specialized accessory functions and tightly regulated half-lives.

Collectively, these emerging examples support the hypothesis that alternative translation initiation represents a biologically meaningful post-transcriptional regulatory mechanism contributing to bacterial proteome complexity. By modulating protein localization, multi-protein complex stoichiometry, and protein stability from a single genetic locus, alternative translation initiation may provide bacteria with a dynamic means of rapidly adapting their proteomes to changing environmental conditions. However, moving the field beyond descriptive, high-throughput multi-omics catalogues towards a mechanistic understanding of Nt-proteoform function requires a shift in experimental strategy. Addressing this challenge will rely on precise endogenous genome-engineering approaches capable of selectively decoupling individual proteoforms within their native genomic context, thereby enabling rigorous functional characterization.

## 7. Conclusions and Future Perspectives

The rapid evolution of bacterial riboproteogenomics is driving a paradigm shift in microbiology, moving genome annotation beyond static gene models toward a dynamic, translatome-aware view of bacterial coding capacity. Increasing evidence suggests that alternative translation initiation may represent a biologically meaningful post-transcriptional regulatory mechanism contributing to bacterial proteome complexity, rather than merely reflecting pervasive translational noise. At the same time, advances in deep-learning and sensitive N-terminomics continue to accelerate the discovery of alternative N-terminal proteoforms, creating a critical bottleneck whereby Nt-proteoform discovery now substantially outpaces their functional characterization. Consequently, the field must progress beyond the systematic cataloguing of alternative translation initiation sites towards defining the physiological roles of individual Nt-proteoforms.

Addressing this challenge will require moving beyond conventional plasmid-based overexpression systems, which may perturb native gene dosage, stoichiometry, and cis-regulatory architecture. Instead, future studies will increasingly rely on precise endogenous genome-engineering approaches, including scarless recombineering and CRISPR/Cas-based technologies, to selectively manipulate individual translation initiation events within their native genomic context. Such approaches will enable functional studies under physiologically relevant conditions and facilitate the establishment of causal relationships between alternative translation initiation and bacterial phenotype.

Combining endogenous genome engineering with proximity-dependent labelling technologies offers a powerful strategy for defining proteoform-specific interactomes, while integrating temporal pulse-labeling with positional proteomics will provide new insights into the effects of alternative N-termini on protein stability and turnover. Ultimately, elucidating how Nt-proteoforms contribute to bacterial physiology, adaptation, and pathogenesis will not only deepen our understanding of prokaryotic proteome complexity but may also reveal novel regulatory mechanisms and potential therapeutic targets.

## Data Availability

No new data were created or analyzed in this study. Data sharing is not applicable to this article.

## Abbreviations

The following abbreviations are used in this manuscript:

6-FT	Six-frame translation
ARF	Allelic replacement frequency
ASC-PCR	Allele-specific colony PCR
aTISs	Alternative translation initiation sites
BASys	Bacterial Annotation System
BV-BRC	Bacterial and Viral Bioinformatics Resource Center
Cas	CRISPR-associated
CDD	Conserved Domain Database
CDS	Coding sequence
COFRADIC	Combined FRActional Diagonal Chromatography
Cos-MAGE	Co-selection MAGE
CRISPR	Clustered Regularly Interspaced Short Palindromic Repeats
CyaA	Calmodulin-dependent adenylate cyclase
dbTIS	Database-annotated translation initiation site
DeepGSR	Deep-learning Structure for the Recognition of Genomic Signals and Regions
DNABERT	DNA-language Bidirectional Encoder Representations from Transformers
DRTs	Defense-associated reverse transcriptases
dsDNA	Double-stranded DNA
*E. coli*	*Escherichia coli*
GeneLM	Gene Language Model
GLIMMER	Gene Locator and Interpolated Markov ModelER
gLM	Genomic language model
gRNA	Guide RNA
HMM	Hidden Markov Model
IB	Inclusion Body
IF2	Initiation Factor 2
iMet	Initiator methionine
INRI-seq	In vitro Ribo-seq
iTIS	Internal translation initiation site
kDa	Kilodalton
LATE	LysN Amino Terminal Enrichment
LC-MS/MS	Liquid Chromatography-Tandem Mass Spectrometry
LgBiT	Large BiT
MAGE	Multiplex Automated Genome Engineering
MetAP	Methionine aminopeptidase
mRNA	Messenger RNA
MS	Mass spectrometry
NAT	N-terminal acetyltransferase
N-terminomics	N-terminal proteomics
Nt-proteoforms	N-terminal proteoforms
OMAR	Oligo-mediated allelic replacement
ORF	Open reading frame
PAM	Protospacer-adjacent motif
PBL	Promiscuous biotin ligase
PDF	Peptide deformylase
PFM	Protein family model
PGAP	Prokaryotic Genome Annotation Pipeline
PPIs	Protein–protein interactions
Prodigal	Prokaryotic Dynamic programming Gene-finding Algorithm
Prokka	Prokaryotic Annotation
pSILAC	Pulse Stable isotope labeling by amino acids in cell culture
RAST	Rapid Annotations using Subsystems Technology
RECKLEEN	Recombineering/CRISPR-based *KLebsiella* Engineering for Efficient Nucleotide editing
REPARATION	RibosomE Profiling Assisted (Re-)AnnotaTION
Ribo-RET	Retapamulin-assisted ribosome profiling
RPM	Reads per million
Ribo-seq	Ribosome profiling
Rubisco	Ribulose-1,5-biphosphate carboxylase/oxygenase
*S*. Typhimurium	*Salmonella enterica* serovar Typhimurium
SD	Shine–Dalgarno
sgRNA	Single guide RNA
sORF	Small open reading frame
SP	Signal peptidase
T3E	Type III secretion system effector
T3SS	Type III secretion system
TAILS	Terminal Amine Isotopic Labeling of Substrates
TetRP	Tetracycline-inhibited ribosome profiling
TIGR	The Institute for Genomic Research
TITER	Translation Initiation Site Detector
TRAINSPOTTER	TRAnslation Initiation SPOTTER
TSS	Transcription start site
